# V3 Loop Truncations in HIV-1 Envelope Impart Resistance to Coreceptor Inhibitors and Enhanced Sensitivity to Neutralizing Antibodies

**DOI:** 10.1371/journal.ppat.0030117

**Published:** 2007-08-24

**Authors:** Meg M Laakso, Fang-Hua Lee, Beth Haggarty, Caroline Agrawal, Katrina M Nolan, Mark Biscone, Josephine Romano, Andrea P. O Jordan, George J Leslie, Eric G Meissner, Lishan Su, James A Hoxie, Robert W Doms

**Affiliations:** 1 Department of Microbiology, University of Pennsylvania School of Medicine, Philadelphia, Pennsylvania, United States of America; 2 Department of Medicine, University of Pennsylvania School of Medicine, Philadelphia, Pennsylvania, United States of America; 3 Department of Microbiology and Immunology, The University of North Carolina, Chapel Hill, North Carolina, United States of America; 4 The Lineberger Comprehensive Cancer Center, The University of North Carolina, Chapel Hill, North Carolina, United States of America; National Institute of Allergy and Infectious Diseases, United States of America

## Abstract

The V1/V2 region and the V3 loop of the human immunodeficiency virus type I (HIV-1) envelope (Env) protein are targets for neutralizing antibodies and also play an important functional role, with the V3 loop largely determining whether a virus uses CCR5 (R5), CXCR4 (X4), or either coreceptor (R5X4) to infect cells. While the sequence of V3 is variable, its length is highly conserved. Structural studies indicate that V3 length may be important for interactions with the extracellular loops of the coreceptor. Consistent with this view, genetic truncation of the V3 loop is typically associated with loss of Env function. We removed approximately one-half of the V3 loop from three different HIV-1 strains, and found that only the Env protein from the R5X4 strain R3A retained some fusion activity. Loss of V1/V2 (ΔV1/V2) was well tolerated by this virus. Passaging of virus with the truncated V3 loop resulted in the derivation of a virus strain that replicated with wild-type kinetics. This virus, termed TA1, retained the V3 loop truncation and acquired several adaptive changes in gp120 and gp41. TA1 could use CCR5 but not CXCR4 to infect cells, and was extremely sensitive to neutralization by HIV-1 positive human sera, and by antibodies to the CD4 binding site and to CD4-induced epitopes in the bridging sheet region of gp120. In addition, TA1 was completely resistant to CCR5 inhibitors, and was more dependent upon the N-terminal domain of CCR5, a region of the receptor that is thought to contact the bridging sheet of gp120 and the base of the V3 loop, and whose conformation may not be greatly affected by CCR5 inhibitors. These studies suggest that the V3 loop protects HIV from neutralization by antibodies prevalent in infected humans, that CCR5 inhibitors likely act by disrupting interactions between the V3 loop and the coreceptor, and that altered use of CCR5 by HIV-1 associated with increased sensitivity to changes in the N-terminal domain can be linked to high levels of resistance to these antiviral compounds.

## Author Summary

The envelope protein of HIV-1 is responsible for binding virus to the surface of cells and mediating viral entry. Viral entry can be prevented by neutralizing antibodies that bind to envelope, and by small molecule inhibitors that bind to viral receptors on the cell surface, such as CCR5. HIV may acquire resistance to these small molecule inhibitors, several of which are being used in clinical trials to treat HIV-infected individuals, through resistance mechanisms that are not well understood. In addition, broadly neutralizing antibodies are rare—the envelope protein possesses structural features that limit antibody binding. We made a partial deletion in a region of envelope that interacts with viral receptors, and which is also widely believed to act as a shield against neutralizing antibodies. Normally, an envelope with such a modification would have total loss of function. However, by passaging virus with the partially deleted envelope in vitro, the envelope acquired adaptive mutations that restored function. Virus with the adapted envelope was highly sensitive to neutralizing antibodies and so may serve as a platform for immunization. This envelope also exhibited complete resistance to small molecule inhibitors that bind to the viral receptor CCR5, and lends insight into a mechanism of drug resistance by which the virus interacts with viral receptors on the cell surface in a novel manner.

## Introduction

The envelope (Env) protein of human immunodeficiency virus type 1 (HIV-1) has an impressive ability to adapt in the face of an evolving immune response, enabling it to avoid recognition by neutralizing antibodies while retaining the ability to mediate viral entry through receptor binding and the induction of membrane fusion [1–3]. Structural features that contribute to immune evasion include an extensive array of N-linked carbohydrate structures that are relatively non-immunogenic, conformational flexibility, and the presence of surface-exposed variable loops that can undergo extensive antigenic variation and still shield more conserved regions of Env that are involved in receptor binding (reviewed in [1]). The V1/V2 variable loop region varies greatly in both amino acid sequence and length [4,5]. Functionally, V1/V2 appears to play a minor role in governing interactions between Env and coreceptors, and its genetic ablation in both HIV-1 and simian immunodeficiency virus is sometimes tolerated without a significant loss of Env function [6–9]. However, genetic removal of the V1/V2 loop is associated with enhanced neutralization of virus by antibodies to the CD4 binding site as well as by antibodies to CD4-induced epitopes that overlap with the conserved coreceptor binding site in the bridging sheet [6,9], a four-stranded anti-parallel beta sheet formed during CD4 binding that connects the inner and outer domains of the gp120 core [10,11].

In contrast to the V1/V2 region, the V3 loop plays an important functional role in viral entry, being the primary determinant of whether CCR5 (R5), CXCR4 (X4), or both CCR5 and CXCR4 (R5X4) can be used as coreceptors to initiate infection (reviewed in [12]). Although the precise mechanism by which the V3 loop governs coreceptor interactions is not clear, sequence motifs commonly associated with CXCR4 usage, including the presence of basic residues at positions 11 and 25 within the V3 loop, have been identified [13,14]. In addition to sequence motifs, the overall length of the V3 loop is likely to be important, as most V3 sequences are 34 or 35 residues in length [15,16]. Structural studies have shown that following CD4 binding, the V3 loop protrudes from the gp120 core by 30 Å, with this length likely enabling it to interact with the viral coreceptor following CD4 binding [17]. The V3 loop is also a target for neutralizing antibodies, and like the V1/V2 region plays a role as an immunological shield, protecting conserved regions in Env that would otherwise be a target for more broadly neutralizing antibodies [6,18,19]. Indeed, genetic truncation of the V3 loop is associated with enhanced binding of antibodies to the CD4 binding site as well as to CD4-induced epitopes [20–22].

Given the functional importance of the V3 loop, it is not surprising that its genetic ablation is usually associated with loss of function [9,20–23]. Recently, we have been able to remove both the V1/V2 region and the V3 loop from a CD4-independent HIV-2 Env through a step-wise process of mutation followed by adaptation, obtaining a replication competent virus (G. Lin, A. Bertolotti-Ciarlet, B. Haggarty, J. Romano, K. McGeehan, et al., unpublished data). In this study, we sought to develop fully functional HIV-1 Envs bearing significant deletions in the V3 loop to probe the role of this domain in protecting virus from neutralization and in coreceptor interactions. We found that one of three HIV-1 Env proteins tested, the R5X4 Env R3A [24,25], could tolerate loss of 15 residues from the distal portion of its V3 loop. Our results show not only that the V3 loop plays an important role in protecting HIV-1 from neutralization, but that altered use of CCR5 by the in vitro–adapted virus results in high levels of resistance to small molecule CCR5 inhibitors, several of which are under clinical development.

## Results

### Derivation of a Replication Competent HIV-1 Strain Containing a 15–Amino Acid Deletion within Its V3 Loop

To produce a replication competent HIV-1 strain lacking much of its V3 loop, we kept the following principles in mind. First, the effects of deletions within the V1/V2 region on Env function are context dependent. In some instances, significant deletions within this region do not affect Env function, whereas in other instances Env function is diminished or lost [6–9]. We reasoned that this might also hold true for truncations within the V3 loop. Therefore, we introduced V3 loop deletions into three different, primary HIV-1 strains: the R5X4 strains R3A and DH12, and the X4 strain TYBE. Second, while previous studies have shown that genetic ablation of the V3 loop typically abrogates Env function, in one instance removal of three amino acids from each arm of the V3 stem from an R5X4 virus resulted in a virus that lost the ability to use CCR5, but could still mediate infection of cells expressing CD4 and CXCR4 [26]. In another study, 23 residues were removed from the center of the 35 amino acid–long V3 loop in the lab-adapted X4 virus strain HXBc2, resulting in an Env protein that could mediate entry into Jurkat cells approximately 10-fold less efficiently than the wild-type (WT) virus Env protein [22]. Finally, we have been able to derive a replication competent HIV-2 from a lab-adapted strain via a process of partial V3 loop deletions followed by viral adaptation in vitro (G. Lin, A. Bertolotti-Ciarlet, B. Haggarty, J. Romano, K. McGeehan, et al., unpublished data). Therefore, we introduced different sized deletions in the V3 loop of each virus strain. Specifically, we removed nine, 15, or 21 residues from the center of the 35 amino acid–long V3 loop, replacing the deleted region with a GAG linker sequence. Equivalent portions in each arm of the V3 loop stem were retained, and this information was used to describe each mutant: ΔV3(12,12) contains the first and last 12 residues of the V3 loop (excluding the Cys residues that mark the beginning and end of the V3 loop) separated by a GAG sequence, ΔV3(9,9) retains the first and last nine residues of the V3 loop separated by a GAG sequence, and ΔV3(6,6) the first and last six residues of the V3 loop separated by a GAG sequence.

Envs containing V3 loop deletions were tested for their ability to mediate cell–cell fusion. We reasoned that Envs unable to mediate cell–cell fusion would also not function in the context of viral infection. To do this, Envs were transiently expressed in quail QT6 cells that were then mixed with QT6 cells expressing CD4 alone, CD4 and CCR5, or CD4 and CXCR4. In this assay, fusion between effector and target cells results in the production of readily quantifiable luciferase activity [27]. We found that DH12 and TYBE Env proteins were rendered non-functional by even the smallest of the V3 loop deletions in this assay (unpublished data). We also found that these Envs lost fusion activity when their V1/V2 loops were removed. In contrast, HIV-1 R3A ΔV3(9,9) (Figure 1A) was able to support cell fusion at levels that were approximately 25% of the WT protein, but only with cells expressing CD4 and CCR5 (Figure 1B). Thus, the ability to utilize CXCR4 appeared to be lost as a result of this V3 loop truncation. Larger deletions in the V3 loop were not tolerated. In contrast, removal of the entire V1/V2 loop from R3A had little effect on membrane fusion activity, and actually enhanced fusion on cells expressing CCR5 (Figure 1B). These results confirm that deletion of the V1/V2 loop on Env function is context dependent, and that significant deletions within the V3 loop can sometimes be tolerated.

**Figure 1:**
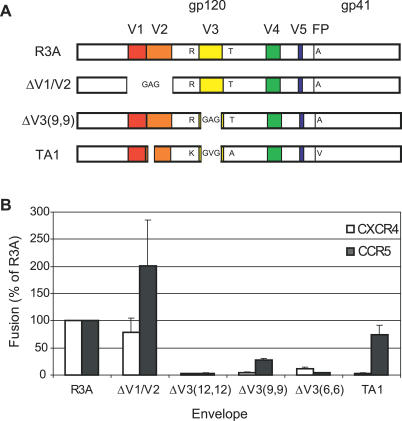
HIV-1 Env Constructs and the Effects of Env Variable Loop Deletions on Fusion Activity (A) A schematic representation of the R3A, ΔV1/V2 (deletion of residues 126–196, but retains the cysteines at the base of the loops), ΔV3(9,9), and TA1 constructs is shown. Amino acid changes in the adapted TA1 Env are indicated, including R254K and T342A in gp120, a mutation in the three amino acid linker introduced into the truncated V3 loop (GAG to GVG), a deletion of residues 185–188 in the V2 loop, and an A to V mutation in the amino terminal residue of gp41. FP, fusion peptide. (B) The effects of Env variable loop deletions on coreceptor use was determined in cell–cell fusion assays, in which cells expressing the indicated Envs were incubated with cells expressing CD4 and CXCR4 (white bars) or CCR5 (black bars). The results are expressed as a percentage of parental R3A Env, and represent the average of 21 independent experiments.

To determine if the reduced cell fusion activity exhibited by ΔV3(9,9) was sufficient to support viral infection, we introduced this Env, as well as the parental R3A Env, into an NL4–3 provirus. Since virus attachment to target cells is often rate-limiting for viral infection in vitro, plasmid DNA was electroporated into SupT1.CCR5.DCSIGNR cells, which stably express CXCR4, CCR5, and the C-type lectin DC-SIGNR. DC-SIGNR mediates efficient attachment of HIV to the cell surface, and thus can boost infection efficiency by accelerating the rate-limiting step of virus attachment [28]. Cells were electroporated twice, then passaged for an additional 7 wk with regular addition of fresh SupT1.CCR5.DCSIGNR cells. After 8 wk of coculture, a spreading infection was established, with over 80% of the cells positive for p24 by immunofluorescence. After 11 subsequent cell-free passages, we compared the replication kinetics of the viral swarm with WT R3A virus on SupT1.CCR5.DCSIGNR cells, and found that the passaged virus replicated with WT kinetics. Thus, we concluded that partial deletion of the V3 loop followed by cell passaging resulted in the acquisition of compensatory mutations that restored Env function to near WT levels, as judged by replication efficiency on SupT1.CCR5.DCSIGNR cells (Figure 2 and unpublished data). Virus with the ΔV1/V2 Env replicated somewhat more slowly than R3A. Bulk sequencing of the ΔV1/V2 virus population did not indicate that any dominant adaptive mutations occurred during passage in tissue culture.

**Figure 2:**
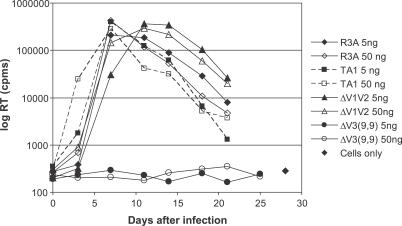
Replication of Virus with R3A Parental, Mutant, and Adapted Envs in Supt1.CCR5.DCSIGNR Cells Cells were infected with 5 or 50 ng of the indicated replication competent virus, and culture supernatants sampled at the times indicated for RT activity. Data are representative of two independent experiments.

### Identification of a Functional Env Protein Bearing a V3 Loop Deletion

Individual *env* clones were obtained from the tissue culture–adapted (TA) viral swarm and tested for functionality in the cell–cell fusion assay. All clones retained the V3(9,9) deletion. We identified a number of Envs that mediated fusion with cells expressing CD4 and CCR5 more efficiently than the parental ΔV3(9,9) Env (clone TA1, Figure 1B). Fusion with cells expressing CD4 and CXCR4 was below the limit of detection for all Envs containing the V3 loop deletion. We introduced one of these tissue culture–adapted *env* clones, TA1, back into a provirus and found that the resulting virus replicated as efficiently as WT R3A virus on SupT1.CCR5.DCSIGNR cells (Figure 2). In contrast, the ΔV3(9,9) virus replicated very poorly.

Sequencing of the TA1 Env as well as four additional Env clones that supported cell fusion with enhanced efficiency relative to the parental, non-adapted ΔV3(9,9) Env revealed two single amino acid changes and a small deletion in the V2 region that were present in all clones, as well as several mutations unique to individual Envs. Together, these common mutations resulted in the loss of an N-linked glycosylation site due to a four–amino acid deletion (residues 185–188) in the V2 region, an A to V mutation in the GAG V3 linker, and a change from a highly conserved A to a V in the N-terminal residue of gp41. In TA1, there were two additional conservative amino acid mutations, R254K in conserved region 2 (C2), and T342A in C3 that resulted in the loss of a second N-linked glycosylation site.

### Tropism of a Virus Bearing a V3 Loop Deletion

Because extensive cell passaging was required to derive virus that could replicate well despite losing much of its V3 loop, it was possible that the adaptation process may have altered viral tropism, and that TA1 might be uniquely adapted to grow on SupT1.CCR5.DCSIGNR cells. We found that in contrast to the WT R3A virus, the TA1 virus was unable to infect cell lines that expressed only CD4 and CXCR4, including SupT1, MT-2, Molt-4 clone 8, and U87.CD4.CXCR4, which is consistent with the failure of TA1 Env to mediate fusion with quail cells expressing CD4 and CXCR4 (Table 1). TA1 was able to mediate infection of cell lines expressing CD4 and CCR5, including SupT1.CCR5 cells lacking DC-SIGNR, Jurkat.tat.CCR5, and U87.CD4.CCR5. We were unable to detect a productive infection in human monocyte-derived macrophages (MDMs) or CD4+ T cells with TA1, whereas the R3A virus can use CCR5 or CXCR4 to enter MDMs, and uses CXCR4 as the preferred coreceptor for entry into CD4+ T cells (Yanji Yi, personal communication).

**Table 1:**
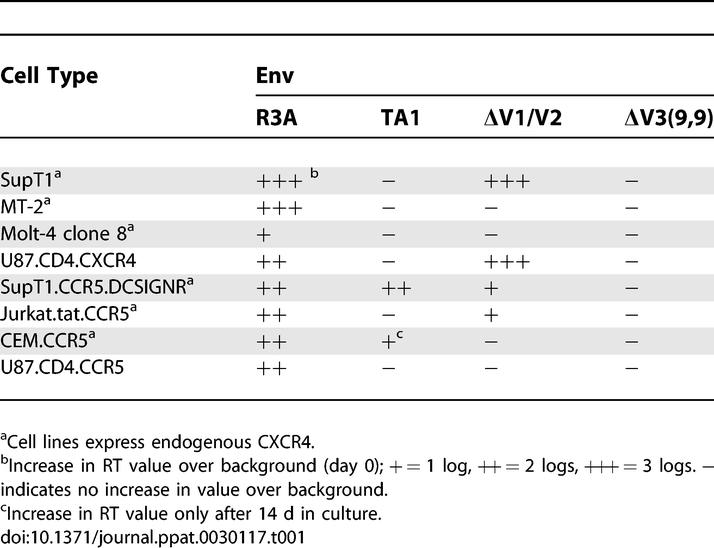
Tropism of Viruses with or without Variable Loop Deletions

Although the ΔV1/V2 Env could mediate fusion with quail QT6 cells expressing either CCR5 or CXCR4, infection with ΔV1/V2-pseudotyped virus or replication competent virus was cell line–dependent. There was robust replication on the CXCR4-expressing cell lines SupT1 and U87.CD4.CXCR4, but no detectable replication in MT-2 or Molt-4 clone 8 cells. Infection of 293.CD4.CCR5 cells, which express low levels of endogenous CXCR4, could be completely blocked by the CCR5 antagonist CMPD167, indicating that only CCR5 was used for entry despite the presence of an alternate coreceptor (unpublished data).

### Functional Analysis of TA1

To study the functional properties of TA1 more carefully, we produced virus pseudotypes so that single cycle viral infection assays could be performed quickly and in a quantitative fashion. Deletions in the V1/V2 region of Env can be associated with enhanced sensitivity of virus to antibody-mediated neutralization [6,9]. To determine if truncations in the V3 loop impacted virus neutralization, we incubated R3A, ΔV1/V2, and TA1 virus pseudotypes with serial dilutions of four different HIV-positive human sera before infecting human 293 cells expressing CD4 and CCR5. As expected, virus pseudotypes bearing the WT R3A Env were neutralized by human sera inefficiently, and then only at relatively high sera concentrations (Figure 3). In contrast, virions bearing the ΔV1/V2 Env were efficiently neutralized by three of the four HIV-positive human sera samples, while virions bearing the TA1 Env were the most neutralization sensitive of all, being efficiently neutralized by all human sera samples tested. To determine if the sensitivity of TA1 virions to neutralization was due to truncation of the V3 loop or due to the presence of the adaptive mutations, we compared the sensitivity of TA1 virions to those bearing the unadapted ΔV3(9,9) truncation. However, since ΔV3(9,9) virions entered cells inefficiently, the viral inoculum had to be increased 5-fold to obtain a sufficiently high signal. When this was done, we found that ΔV3(9,9) virions were also neutralization sensitive, though not quite to the same extent as TA1 virions (Figure 3D).

**Figure 3:**
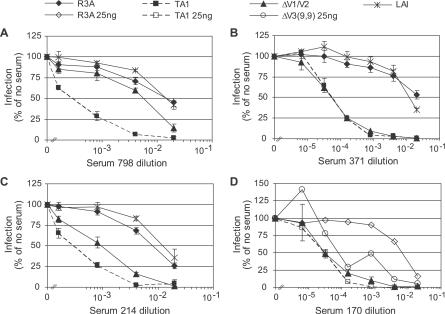
Sensitivity of Parental, Mutant, and Adapted Envs to Neutralization by HIV Patient Sera (A–D) Serum from four HIV-infected individuals who were not on antiretroviral therapy and had CD4 counts >400 were tested for neutralization activity by limiting dilution. Virus pseudotypes bearing the indicated Env were incubated with serial dilutions of patient sera for 1 h, added to 293.CD4.CCR5 cells, and the amount of luciferase activity measured 3 d later. The amount of luciferase activity obtained for each virus pseudotype in the absence of serum was set to 100%. The results shown are the average for three or four independent experiments, ± standard error of the mean.

To evaluate the mechanism underlying the markedly enhanced sensitivity of TA1 to neutralization by HIV-positive human sera, we performed neutralization assays with several well-defined, broadly neutralizing monoclonal antibodies (MAbs). MAb IgG b12, which binds to gp120 and prevents subsequent binding to CD4 [29,30], neutralized TA1 approximately 100-fold more efficiently than it neutralized R3A, ΔV1/V2, or the lab-adapted X4 virus strain LAI (Figure 4A). MAb 4E10, which binds to an epitope in the membrane proximal region of gp41 [31], failed to neutralize R3A efficiently, but exhibited modestly enhanced neutralization activity against both TA1 and ΔV1/V2 (Figure 4B). We also tested the ability of MAb 17b to neutralize our panel of viruses. MAb 17b binds to a CD4-induced epitope in gp120 that includes a portion of the bridging sheet [32]. Although antibodies to this region appear to be relatively common in sera from HIV-infected individuals, they fail to neutralize primary HIV-1 isolates [33]. However, viruses lacking the V1/V2 loops are susceptible to 17b-mediated neutralization, as are CD4-independent HIV-1 strains in which the 17b epitope is present in the absence of CD4 binding [6,9,34]. We found that 17b failed to neutralize R3A or LAI, but did neutralize ΔV1/V2. Moreover, TA1 was at least 100-fold more sensitive to neutralization by 17b than the ΔV1/V2 virus (Figure 4C). Thus, truncation of the V3 loop rendered HIV-1 R3A Env extremely sensitive to antibody mediated neutralization, with antibodies to the CD4 binding site and the bridging sheet in gp120 in particular showing markedly enhanced neutralization activity against this Env.

**Figure 4:**
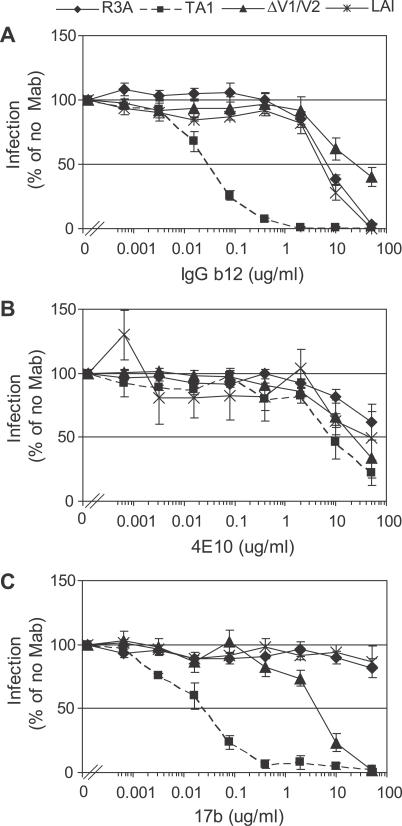
Sensitivity of Viruses to Neutralization by MAbs Luciferase reporter viruses pseudotyped with parental, mutant, or adapted Envs as indicated were incubated with various concentrations of the MAbs (A) IgG b12, (B) 4E10, or (C) 17b for 1 h prior to infection of 293.CD4.CCR5 cells. The amount of luciferase activity obtained for each virus pseudotype in the absence of any antibody was set to 100%. The results shown are the average for three independent experiments, ± standard error of the mean.

### Sensitivity of TA1 Virus to Entry Inhibitors

Since the V3 loop plays an important role in mediating interactions between Env and coreceptors, we hypothesized that truncating this functionally important region could influence viral sensitivity to entry inhibitors. We have found that similar deletions of the V3 loop in a dual-tropic, lab-adapted HIV-2 isolate confers resistance to both R5 and X4 antagonists (G. Lin, A. Bertolotti-Ciarlet, B. Haggarty, J. Romano, K. McGeehan, et al., unpublished data). Compared to the parental R3A virus, we found that virus pseudotypes bearing the ΔV1/V2 Env protein were approximately 5-fold more sensitive to inhibition by the fusion peptide inhibitor enfuvirtide, while the TA1 virus was even more sensitive (Figure 5A). Changes in gp120 can influence sensitivity to enfuvirtide, with one mechanism being the alteration of the rate of membrane fusion [35,36]. Thus, it is possible that loss of the entire V1/V2 loop or a portion of the V3 loop could reduce the rate of Env-mediated membrane fusion by reducing affinity for coreceptor and/or the efficiency of gp41 triggering, resulting in prolonged exposure of the enfuvirtide binding site in the HR1 region of gp41.

**Figure 5:**
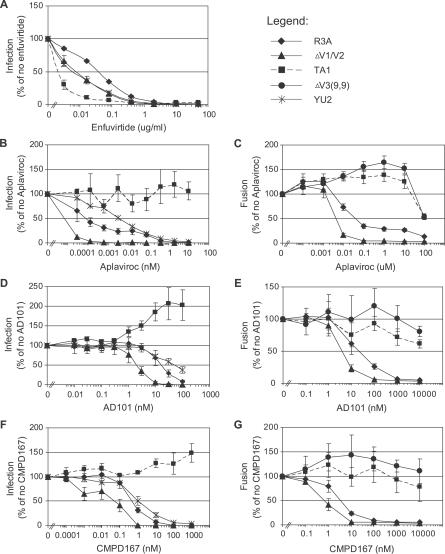
Relative Sensitivity of Parental, Mutant, and Adapted Envs to Entry Inhibitors Pseudotyped luciferase reporter viruses were used to determine sensitivity to the fusion inhibitor (A) enfuvirtide, or the CCR5 inhibitors (B) aplaviroc, (D) AD101, and (F) CMPD167. Sensitivity of Envs to (C) aplaviroc, (E) AD101, and (G) CMPD167 was determined in a cell–cell fusion luciferase reporter assay, using QT6 effector cells expressing the indicated Env and QT6 target cells expressing CD4 and CCR5. Results are the average of three or four independent experiments, ± standard error of the mean.

We also tested the sensitivity of TA1 and ΔV1/V2 to three different small molecule CCR5 inhibitors (CMPD 167, aplaviroc, and AD101) using human 293.CD4.CCR5 cells as targets. When used singly, these compounds inhibited R3A infection by approximately 80%, with the residual infectivity resulting from the presence of endogenous, low levels of CXCR4 expressed on the human 293 cells that could be utilized by this R5X4 virus. This CXCR4- dependent entry could be efficiently blocked by the small molecule inhibitor AMD3100 (unpublished data). Thus, a high concentration of AMD3100 was included to suppress this background level of infection when the sensitivity of R3A virions to CCR5 inhibitors was being determined.

Virus pseudotypes bearing the ΔV1/V2 Env protein used CCR5 to enter 293.CD4.CCR5 cells, and were 5- to 10-fold more sensitive than the parental R3A to inhibition by the panel of CCR5 inhibitors (Figure 5B, 5D, and 5F). In contrast, TA1 virus pseudotypes were completely resistant to the CCR5 inhibitors. Infection was not observed in the absence of CCR5, confirming that TA1 infection was CCR5-dependent (unpublished data). Interestingly, infection was consistently enhanced in the presence of high levels of AD101, though not by aplaviroc. A small degree of enhancement was also observed when very high levels of CMPD167 were used. These results suggest that TA1 utilizes the AD101-bound conformation of CCR5 more efficiently than CCR5 that is not bound to this allosteric inhibitor. We also performed cell–cell fusion assays (in the absence of AMD3100) and found that TA1-mediated cell–cell fusion could only be reduced by 50% at very high levels of aplaviroc (Figure 5C). Fusion mediated by the parental ΔV3(9,9) Env was also resistant to CCR5 inhibitors, indicating that truncation of the V3 loop alone imparted resistance to this class of entry inhibitors (Figure 5C, 5E, and 5G). Together, these results show that a significant truncation in the V3 loop restricts the tropism of an R5X4 Env such that its fusion activity is CCR5-dependent, greatly enhances sensitivity to neutralization by human sera and some MAbs, and results in very high levels of resistance to CCR5 inhibitors.

### Altered Use of CCR5 by TA1

The high level of resistance exhibited by TA1 to CCR5 inhibitors suggests that it can efficiently utilize the drug-bound conformation of the coreceptor. If so, TA1 may interact with CCR5 differently than the parental R3A Env. A model for Env–CCR5 interactions is that the V3 loop interacts with the extracellular loops (ECLs) of the receptor, particularly ECL2, while the amino terminal domain of CCR5 interacts with the bridging sheet and base of the V3 loop [17,37]. We hypothesized that the shortened V3 loop would result in a weaker interaction with CCR5 ECL2, and that to compensate for this, TA1 would have increased reliance on the CCR5 N-terminus for entry. To test this hypothesis, we first used MAbs to the CCR5 N-terminus and ECL2 in neutralization assays. TA1, but not R3A, was highly sensitive to neutralization by MAb CTC5 (Figure 6A). CTC5 binds to an epitope in the distal N-terminus of CCR5, and does not normally prevent infection by HIV-1 [38]. MAb 2D7, which binds to CCR5 ECL2 and prevents infection by most HIV strains [38,39], blocked entry of both TA1 and R3A (unpublished data).

**Figure 6:**
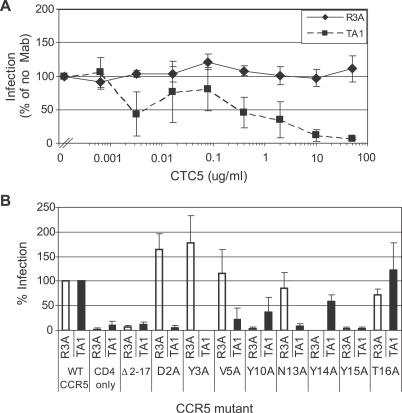
Altered Dependence on CCR5 N-Terminus for Viral Entry (A) Luciferase reporter viruses pseudotyped with R3A or TA1 were incubated with various concentrations of the MAb CTC5 for 1 h prior to infection of 293.CD4.CCR5 cells. The amount of luciferase activity obtained for each virus pseudotype in the absence of any antibody was set to 100%. The results shown are the average for three independent experiments, ± standard error of the mean. (B) Luciferase reporter viruses pseudotyped with R3A (white bars) or TA1 (black bars) Env were incubated with 293T cells expressing CD4 only, CD4 and CCR5, or CD4 and mutant CCR5, as indicated. Δ2–17 refers to a CCR5 construct lacking amino acids 2–17. Single amino acid mutants refer to changes in the CCR5 N-terminus. The amount of luciferase activity obtained for each virus pseudotype on WT CCR5 was set to 100%. The results shown are the average for three independent experiments, ± standard error of the mean.

To more finely map the residues in the CCR5 N-terminus required for TA1 entry, we used a series of CCR5 mutants in which single amino acids were changed to alanine. We concentrated on acidic residues and tyrosines, as sulfation of tyrosines is important for coreceptor function [40]. 293T cells were transiently transfected with expression plasmids for CD4 alone, or CD4 and either WT CCR5 or a CCR5 mutant. Cells were infected the following day, and luciferase assays performed 3 d post-infection. Changes in the tyrosine residues at positions 10, 14, and 15 resulted in nearly complete ablation of viral entry by R3A and TA1 (Figure 6B). However, TA1 entry was much more sensitive to changes at the extreme amino terminus of CCR5, particularly D2A and Y3A, confirming the results of the neutralization assay with CTC5, whose epitope overlaps these amino acids. Thus, TA1 appears to interact with CCR5 in a manner that is distinct from the parental R3A Env, in that it is highly sensitive to changes in the distal portion of the CCR5 N-terminus and to neutralization by a MAb that binds to this region.

## Discussion

While the V3 loop of HIV Env proteins exhibits extensive sequence variability, it nonetheless faces significant functional and structural constraints. The V3 loop is the major determinant of coreceptor choice, with changes in specific residues being associated with alterations in viral tropism at the level of CCR5 and CXCR4 recognition, as well as with viral sensitivity to chemokines and entry inhibitors [35,41–46]. In addition, it is a target for neutralizing antibodies, and may help to shield the conserved domains on the gp120 core from what would otherwise be broadly neutralizing antibodies (reviewed in [12,47]). Structurally, the V3 loop exhibits little variation in length, with the vast majority of isolates containing V3 loops that contain 34 or 35 residues [15,16]. This conservation of length may be related to the requirement that the V3 loop engage CCR5 or CXCR4 only after Env has bound CD4, which might position gp120 a defined distance from the cell surface and, as a result, from the coreceptors to which it must bind. The highly conserved length of the V3 loop stands in marked contrast to the V1/V2 and V4 variable loops, which exhibit extensive sequence and length variation and play a subsidiary role in viral entry.

In light of these constraints, it is perhaps not surprising that truncations in the V3 loop are associated with either lost or significantly reduced Env function [21,26]. In this report, we introduced significant V3 loop truncations into three HIV-1 Env proteins, of which only one (R3A) retained functional competence. Until a larger number of Envs are examined, it is not possible to predict whether any given virus will be able to tolerate significant deletions within the V3 loop. R3A was derived from an HIV-positive individual who progressed to AIDS rapidly, with syncytium-inducing viruses being detected early after infection [48]. Virus bearing the R3A Env is dual-tropic and is unusually cytopathic in human fetal thymus organ cultures and in SCID-hu Thy/Liv mice [49,50]. R3A can also mediate fusion with SupT1 cells very efficiently, with this property being due at least in part to the V1/V2 region [24]. Perhaps because of its highly fusogenic nature, R3A was better able to tolerate a significant deletion within its V3 loop.

Truncation of the V3 loop on R3A was associated with markedly enhanced sensitivity of virus to neutralization by MAbs directed to the bridging sheet and CD4 binding site (but not to other epitopes), as well as to neutralization by HIV-positive human sera samples that were otherwise unable to neutralize the parental virus with an intact V3 loop. These findings are reminiscent of observations that CD4-independent HIV-1 and simian immunodeficiency virus strains are highly neutralization sensitive [7,34,51–53]. CD4-independent viruses are thought to exist in a conformation that resembles the CD4-bound conformation. As a result, MAbs to the bridging sheet can bind to these viruses in the absence of CD4, resulting in efficient neutralization [34]. The fact that CD4-independent viruses are easily neutralized by HIV-positive human sera suggests that antibodies to the bridging sheet are generated during the course of viral infection, but cannot bind to and neutralize WT virus [33,52]. Our results support the widely accepted view that the V3 loop plays an important role in protecting this conserved domain from antibody recognition. However, TA1 is CD4-dependent. Thus, upon truncation of the V3 loop, it is possible that antibodies to the bridging sheet or other conserved domains on the gp120 core can bind to Env after the virus has bound CD4 at the cell surface, which suggests that the V3 loop may function in part as a steric shield, protecting conserved domains in gp120 from circulating antibodies even after the virus has engaged CD4.

The most striking result of our study was that the TA1 virus was completely resistant to three different CCR5 inhibitors. In contrast, the use of CCR5 by the parental R3A Env could be efficiently blocked by CCR5 inhibitors, while the ΔV1/V2 virus exhibited enhanced sensitivity to these compounds. We hypothesize that loss of the V1/V2 region is associated with reduced affinity for CCR5, as this could account not only for the fact that the ΔV1/V2 virus is more sensitive than R3A to CCR5 inhibitors, but that it exhibits enhanced sensitivity for enfuvirtide as well. The antiviral activity of enfuvirtide is subject to kinetic constraints, as the binding site for this drug in the HR1 region of gp41 becomes available only after Env has engaged CD4 [35,54]. Thus, there is a kinetic window between CD4 binding and membrane fusion during which Env is sensitive to enfuvirtide. All other things being equal, reduced affinity for coreceptor slows membrane fusion kinetics, prolongs the exposure of the enfuvirtide binding site, and enhances sensitivity to enfuvirtide. In addition, reduction in CCR5 affinity in genetically similar Envs increases sensitivity to CCR5 inhibitors [55].

High-level resistance of HIV-1 isolates to CCR5 inhibitors is unusual, though there is baseline variability in the sensitivity of R5 Env and R5X4 Envs to these antiviral compounds [56–58]. When resistance is selected for in vitro, it has often been associated with continued use of CCR5 by the adapted virus rather than a “coreceptor switch” in which the ability to use CXCR4 is acquired [43,46,59]. It has been hypothesized that the small molecule CCR5 inhibitors operate via an allosteric mechanism, binding to and stabilizing CCR5 conformations that are not recognized by HIV-1 Env [60,61]. Resistance to CCR5 inhibitors often, but not always, involves changes in the V3 loop [45,59,62], enabling virus to utilize the drug-bound conformation of the receptor. Whether this entails recognition of altered CCR5 conformations or regions of CCR5 that are not conformationally altered by the drug, increased affinity for CCR5, or a combination of mechanisms, is not clear.

How might the complete resistance of TA1 to multiple CCR5 inhibitors be explained, and could this provide insight into how HIV acquires resistance to these antiviral agents in vivo? It is evident that virus bearing the TA1 Env requires CCR5 to infect cells. Thus, its resistance to CCR5 inhibitors cannot be ascribed to the use of CXCR4 or alternative coreceptors. Rather, TA1 continues to use CCR5 efficiently, even in the face of very high concentrations of potent CCR5 inhibitors, which indicates that TA1 recognizes the drug-bound conformation of CCR5. In fact, TA1 may recognize the drug-bound conformation of CCR5 more efficiently than the unbound receptor, as we consistently observed enhanced fusion and infection of CCR5-positive cells by TA1 in the presence of high concentrations of the CCR5 inhibitor AD101 and, to a lesser extent, in the presence of CMPD167 and aplaviroc. A variety of studies suggest that the V3 loop contacts the extracellular loops of CCR5, with the second extracellular loop being particularly important for coreceptor function (reviewed in [12,63]). CCR5 inhibitors are thought to bind to a hydrophobic cavity formed by the first, second, third, and seventh transmembrane domain helices [64], and may disrupt subsequent binding by MAbs that recognize epitopes in the second extracellular loop of the receptor [61]. Thus, CCR5 inhibitors may alter the conformation of the extracellular loops of the receptor, preventing interactions with the V3 loop [65]. Our results indicate that, faced with the loss of the V3 loop, the TA1 virus acquired adaptive changes that enhanced its ability to interact with the N-terminal domain of CCR5, a region of the receptor that is thought to contact the bridging sheet of gp120 and the base of the V3 loop and whose conformation may not be greatly affected by CCR5 inhibitors. If such a “shift” in how Env recognizes CCR5 is typically associated with inhibitor resistance, then the identification of inhibitors that perturb the amino terminal domain of CCR5 may prove to be useful antiviral agents, with viruses such as TA1 being receptor ligands that can be used to identify such compounds.

## Materials and Methods

### Cells.

QT6, 293T/17, and 293.CD4.CCR5 cell lines were cultured in DMEM supplemented with 10% fetal bovine serum, 60 μg/ml penicillin, and 100 μg/ml streptomycin (complete DMEM), plus 0.3 mg/ml G418 and 0.2 mg/ml zeomycin where appropriate. U87.CD4, U87.CD4.CCR5, and U87.CD4.CXCR4 cell lines were cultured in complete DMEM plus 0.3 mg/ml G418, and 1 μg/ml puromycin for cells expressing coreceptor. SupT1, SupT1.CCR5.DCSIGNR, MT-2, Molt-4 clone 8, and Jurkat.tat.CCR5 cells were cultured in RPMI1640 supplemented with 10% fetal bovine serum, 60 μg/ml penicillin, and 100 μg/ml streptomycin (complete RPMI). SupT1.CCR5.DCSIGNR cells were made by transfecting SupT1 cells with pCDNA3.1-CCR5 and selecting in 1 μg/ml puromycin. The puromycin-resistant cell population was transduced with a VSV G–pseudotyped retrovirus vector, Lenti6-DCSIGNR, and selected in 10 μg/ml blasticidin for 14 d.

### Plasmids.

All viral *env* genes used for production of virus pseudotypes, including 400 nt upstream of the *env* initiation codon with the complete coding sequences of *tat, rev,* and *vpu,* were cloned into the XhoI and EcoRI sites of pHSPG [24]. All viral *env* genes used for fusion assays were TOPO cloned into pcDNA3.1 (Invitrogen, http://www.invitrogen.com/). The QuikChange Site-Directed Mutagenesis Kit (Stratagene, http://www.stratagene.com/) was used to construct variable loop deletion mutants. Human CD4, CXCR4, and CCR5 in pcDNA3.1 and the reporter plasmid encoding luciferase under the control of a T7 promoter (pT7.luciferase) used in cell–cell fusion assays have been described previously [27].

R3A was cloned from a rapid progressor with early loss of T cell homeostasis [25]. It allows entry into both macrophages and T cells, as well as into both CXCR4- and CCR5-expressing cell lines. DH12 was isolated from an AIDS patient in a screen to identify primary viruses that could spread with rapid kinetics in chimpanzee PBMCs; it allows entry into human monocyte-derived macrophages and human T cell lines [66]. The primary isolate TYBE is an X4-tropic isolated from the cerebrospinal fluid cell pellet of an individual with AIDS [67].

### Cell–cell fusion assay.

This assay has been previously described in detail [27]. Briefly, “target” QT6 cells were cotransfected with CD4 and a coreceptor or control expression plasmid as well as with a luciferase reporter gene expression plasmid under the control of a T7 promoter (pGEM2 T7-luc; Promega, http://www.promega.com/). QT6 “effector” cells were transfected with Env expression plasmids and infected with a recombinant vaccinia virus expressing T7 polymerase (vTF1.1) [68]. Effector cells were added to target cells approximately 18 h post-transfection and the cells allowed to interact at 37 °C for a period of 7–8 h. Cells were lysed in 0.5% Triton X-100 in PBS, luciferase substrate was added, and luciferase activity was measured in a luminometer [69]. The sensitivity of Env-mediated fusion to aplaviroc, enfuvirtide, CMPD167, and AD101 was determined by fusing cells in the presence of increasing amounts of inhibitors, as previously described [35,70].

### Adaptation of the TA1 Env.

Viruses with adapted Envs were generated by electroporating (250 V; 950 μF) SupT1.CCR5.DCSIGNR cells (5 × 10^6^ cells in 4-mm cuvettes) with 20 μg of pNL-R3A ΔV3(9,9). Viral growth was monitored by immunofluorescence microscopy using antibodies to p24Gag, and cell-free passage of virus onto uninfected SupT1.CCR5.DCSIGNR cells was performed when cultures were > 80% for p24 expression. Briefly, a Shandon Cytospin (1,500 rpm × 5 min; Thermo Fisher Scientific, http://www.thermo.com/) was used to gently affix cells to a glass slide. Cells were fixed for 10 min in methanol:acetone, then dried and stained with anti-p24 murine MAb (25.4 provided by Jan McClure, University of Washington) followed by a FITC-conjugated anti-mouse immunoglobulin. After 8 wk of coculture with uninfected cells, >80% of cells were positive by immunofluorescence assay. Supernatant was harvested and used to infect naive SupT1.CCR5.DCSIGNR cells. This first cell-free passage was designated Passage 1. Cell-free supernatant was passaged on uninfected cells weekly for 26 wk. To derive *env* clones from infected cells, genomic DNA was prepared with QIAamp DNA Mini kit according to the manufacturer's instructions (Qiagen, http://www.qiagen.com/), and *env* sequences were PCR amplified from genomic DNA and TOPO cloned into pCR2.1 (Invitrogen). PCR-amplified *envs* from proviral sequences were sequenced in bulk for selected passages. Individual *envs* were cloned after 11 cell-free passages.

### Viral infection assays.

Luciferase reporter pseudotype viruses were produced in 293T cells by cotransfection with the NL4–3-based luciferase vector, pNL-luc (E^−^, Vpr^−^), and LAI, YU2, R3A, or R3A mutant envelope expression plasmids as previously described [71,72]. Replication competent viruses were produced in 293T/17 cells by transfection of an NL4–3-based proviral vector with the HIV *env* cloned into the XhoI and EcoRI sites. Viral titer was normalized by p24 value. For neutralization sensitivity assays, 5 or 10 ng of virus was incubated with serial dilutions of the MAb or patient serum for 1 h at 37 °C, and spinoculated onto 293.CD4.CCR5 cells [73]. Cells were subsequently incubated at 37 °C for 3 h, then aspirated and re-fed with complete DMEM. Cells were assayed for luciferase expression 3 d post-infection. Sera from HIV patients were obtained from the Clinical Core of the University of Pennsylvania Center for AIDS Research; all patients had CD4 counts >400/μl. MAbs 17b, IgG b12, and 2D7 were obtained from the NIH AIDS Research and Reference Reagent Program (https://www.aidsreagent.org/); 4E10 was obtained from the International AIDS Vaccine Initiative Neutralizing Antibody Consortium Repository; and CTC5 was obtained from R&D Systems (http://www.rndsystems.com/). For drug inhibition assays, 5 or 10 ng of virus was incubated with enfuvirtide for 1 h before spinoculation. The CCR5 antagonists, aplaviroc (GlaxoSmithKline, http://www.gsk.com/), AD101 (Schering-Plough, http://www.sch-plough.com/), and CMPD167 (Merck Research Laboratories, http://www.merck.com/), were incubated with target cells in the presence of 100 nM AMD3100 (NIH AIDS Research and Reference Reagent Program) for 1 h at 37 °C before addition of virus, followed by spinoculation. Cells were subsequently incubated with virus and drug for 3 h at 37 °C, then the medium was replaced with complete DMEM; cells were assayed for luciferase expression 3 d post-infection [70].

### Growth curves and determination of envelope tropism.

Viral replication in cell lines was quantified by inoculating SupT1.CCR5.DCSIGNR cells with 5 or 50 ng of replication competent virus and determining reverse transcriptase activity in a ^3^H-thymidine-based assay on culture supernatants pelleted by ultracentrifugation [74]. For host range studies, 5 and 50 ng of replication competent virus was used to infect CCR5- and CXCR4-expressing cell lines, and reverse transcriptase activity was quantified at days 0, 7, and 14.

## Supporting Information

### Accession Numbers

Nucleotide sequences for clones TA1, ΔV1/V2, and ΔV3(9,9) were submitted to GenBank (http://www.ncbi.nlm.nih.gov/Genbank/index.html) under accession numbers EF613594–EF613596.

## Abbreviations

Env: envelope
HIV-1: human immunodeficiency virus type 1
MAb: monoclonal antibody
WT: wild-type
